# Antibacterial Effects of the Essential Oils of Commonly Consumed Medicinal Herbs Using an *In Vitro* Model

**DOI:** 10.3390/molecules15117532

**Published:** 2010-10-27

**Authors:** Marina Soković, Jasmina Glamočlija, Petar D. Marin, Dejan Brkić, Leo J. L. D. van Griensven

**Affiliations:** 1 Plant Research International, Wageningen University and Research Centre, Droevendaalsesteeg 1, 6708PB Wageningen, The Netherlands; 2 Institute for Biological Research “Siniša Stanković”, Bulevar Despota Stefana 142, University of Belgrade, 11000 Belgrade, Serbia; 3 Institute of Botany, Faculty of Biology, University of Belgrade, Stud. trg 16, 11000 Belgrade, Serbia; 4 Les Laboratoires Servier, Bulevar Mihajla Pupina 165v, 11 070 Novi Beograd, Serbia

**Keywords:** disc-diffusion, essential oils, food spoilage bacteria, herbs, human pathogens, microdilution method, natural antimicrobial agents, structure-activityIntroduction

## Abstract

The chemical composition and antibacterial activity of essential oils from 10 commonly consumed herbs: *Citrus aurantium, C. limon, Lavandula angustifolia, Matricaria chamomilla, Mentha piperita, M. spicata, Ocimum basilicum, Origanum vulgare, Thymus vulgaris* and *Salvia officinalis* have been determined. The antibacterial activity of these oils and their main components; *i.e. *camphor, carvacrol, 1,8-cineole, linalool, linalyl acetate, limonene, menthol, α-pinene, β-pinene, and thymol were assayed against the human pathogenic bacteria *Bacillus subtilis, Enterobacter cloacae, Escherichia coli *O157:H7, *Micrococcus flavus, Proteus mirabilis, Pseudomonas aeruginosa, Salmonella enteritidis, S. epidermidis, S. typhimurium*, and *Staphylococcus aureus*. The highest and broadest activity was shown by *O. vulgare* oil. Carvacrol had the highest antibacterial activity among the tested components.

## 1. Introduction

Food processors, food safety researchers, and regulatory agencies have been increasingly concerned with the growing number of food-borne illness outbreaks caused by pathogens like *Staphylococcus aureus*, *Salmonella *sp*.*, *Clostridium perfringens*, *Campylobacter*, *Listeria monocytogenes*, *Vibrio parahaemolyticus*, *Bacillus cereus*, and entero-pathogenic *Escherichia coli* [1,2]. These bacteria cause over 90% of all cases of food poisoning. 

Infections due to bacterial species also remain a serious clinical problem. Emerging resistance of bacterial species is seriously decreasing the number of effective antimicrobials. Because of increasing pressure of consumers and legal authorities, the food industry has tended to reduce the use of chemical preservatives in their products to either completely nil or to adopt more natural alternatives for the maintenance or extension of product shelf life [3].

Plants and their essential oils are potentially useful sources of antimicrobial compounds. Numerous studies have been published on the antimicrobial activities of plant compounds against many different types of microbes, including food-borne pathogens [2,4,5,6,7]. The main constituents of essential oils – mono- and sesquiterpenes including carbohydrates, phenols, alcohols, ethers, aldehydes and ketones – are responsible for the biological activity of aromatic and medicinal plants as well as for their fragrance. Due to these properties, spices and herbs have been added to food since ancient time, not only as flavouring agents but also as preservatives [8].

The general objective of the present study was to test a broad variety of naturally occurring and potentially food-compatible essential oils and oil compounds commonly used in herbal drinks for their antimicrobial potential against an epidemiologically relevant group of bacterial food-borne pathogens.

## Results and Discussion

The results of the chemical analyses of the essential oils of the different herbs are presented in Table 1. The yield of *M*. *piperita *oil is 3.2% (v/w), and its main components are menthone (12.7%), menthol (37.4%) and methyl acetate (17.4%). The yield of *Mentha spicata *oil is 1.5% (v/w), and the main components are menthone (21.9%) and carvone (49.5%). Limonene is the most abundant component in *C*. *aurantium *(90.0%) and *Citrus limon *(59.7%) oils. The yield of *Matricaria chamomilla *oil is 0.7% (v/w), and trans-β-farnesene is the major component (43.5%). Linalool (27.2%) and linalyl acetate (27.5%) are the most abundant components in *Lavandula angustifolia *oil (yield is 3% (v/w). Linalool is also the main component in *Ocimum basilicum *oil with 69.3 % (yield is 0.5% (v/w). Camphor (16.7%) and α-thujone (31.7%) are the main components in *Salvia officinalis *oil (yield is 2.2% (v/w). The yield of *Origanum vulgare *oil is 1.5% (v/w), and carvacrol (64.5%) is the dominant component. The yield of *Thymus vulgaris *oil is 3% (v/w), and the major components are *p*-cymene (19.0%) and thymol (64.5%).

**Table 1:** Chemical composition of the essential oils of commonly consumed herbs.

Components	*M.s. * %	*M.p*. %	*C.l. * %	*C.a. * %	*M.c. * %	*L.a. * %	*O.b.* %	*S.o.* %	*O.v.* %	*T.v.* %	*RI*
Tricyclene	0.3	-	0.4	-	0.2	0.1	-	-	-	-	926
α-Thujene	0.1	-			-	0.6	-	0.1	2.0	1.2	931
α-Pinene	0.1	-	2.9	-	-	0.2	0.1	4.8	-	1.2	939
Camphene	-	-	-	-	0.1	-	0.1	6.9	-	0.8	948
Sabinene	0.7	2.5	-	-	0.4	-	-	0.1	2.2	0.6	973
β-Pinene	0.4	-	17.3	-	-	-	-	1.7	-	0.4	980
β-Myrcene	2.3	0.5	1.72	-	-	-	0.3	1.1	-	1.0	991
3-Octanol	-	0.1			-	-	-	-	-	-	993
δ-3-Carene	-	-	-	6.2	-	-	-	-	2.2	-	1011
α-Terpinene	-	0.1	-	-	0.1	0.3	0.1	-	-	0.6	1018
*p*-Cymene	0.5	0.1	-	-	0.2	-	-	1.0	10.9	19.0	1026
Limonene	5.7	6.9	59.7	90.0	0.2	8.5	1.0	2.6	-	0.5	1030
1,8-Cineole	3.0	5.6	-	-	0.4	3.3	0.8	8.7	-	0.8	1031
*cis*-Ocimene	-	0.1	0.1	-	1.7	-	0.1	-	-	-	1040
*trans*-Ocimene	-	0.2	-	-	1.9	-	0.5	-	-	1.3	1050
γ-Terpinene	1.3	0.3	11.2	-	0.1	-	-	0.4	10.8	4.0	1068
*cis*-Linalool oxide	-	-	-	-	-	2.4	-	-	-	-	1072
Fenchone	-	-	-	-	-	0.6	-	-	-	-	1087
α-Terpinolene	0.3	0.1	0.3	-	-	-	0.4	0.3	-	-	1088
*trans*-Sabinene hydrate	-	-	-	-	0.3	-	-	-	-	-	1097
Linalool	-	0.2	-	-	-	27.2	69.3	-	-	0.7	1098
α-Thujone	-	-	-	-	-	-	-	31.7	-	-	1102
*endo*-Fenchol	-	-	-	-	-	0.1	-	-	-	-	1112
β-Thujone	-	-	-	-	-	-	-	4.6	-	-	1114
*iso*-3-Thujanol	-	-	0.2	-	-	-	-	-	-	-	1133
*trans*-Limonene oxide	-	-	0.2	-	-	-	-	-	-	-	1137
Camphor	-	-	-	-	-	1.1	0.3	16.7	-	0.2	1143
Menthone	21.9	12.7			-	-	-	-	-	-	1154
Menthofuran	-	6.8			-	-	-	-	-	-	1164
Borneol	-	-	-	-	-	2.5	0.3	2.6	-	1.7	1165
Menthol	0.5	37.4			-	-	-	-	-	-	1173
Terpin-4-ol	0.7	-	-	-	-	2.1	-	0.4	-	1.8	1177
α-Terpineol			0.3			4.2	0.6	0.1	-	-	1189
*cis*-Dihydrocarvone	0.3	-	-	-	-	-	-	-	-	-	1193
Methyl chavicol	-	-	-	-	-	-	2.4	-	-	-	1195
*trans*-Dihydrocarvone	0.5	-	-	-	-	-	-	-	-	-	1200
*trans*-Carveol	0.2	-	-	-	-	-	-	-	-	-	1217
Nerol	-	-	-	-	-	-	0.4	-	-	-	1228
Thymol methyl ether	-	-	-	-	-	-	-	-	-	0.2	1235
Neral	-	-	0.8	-	-	-	-	-	-	-	1240
Carvone	49.5	-	-	-	-	-	0.1	-	-	-	1242
Pulegone	-	1.2	-	-	-	-	-	-	-	-	1243
Carvacrol methyl ether	-	-	-	-	-	-	-	-	-	1.7	1244
Piperitone	0.6	0.8	-	-	-	-	-	-	-	-	1252
Geraniol	-	-	-	-	-	-	1.9	-	-	-	1253
*trans*-Anethole	0.5	-	-	-	-	-	-	-	-	-	1283
Linalyl acetate	-	-	-	-	-	27.5	-	-	-	-	1257
Bornyl acetate	-	-	-	-	-	0.1	0.3	-	-	-	1285
Lavandulyl acetate	-	-	-	-	-	6.6	-	-	-	-	1289
Thymol	-	-	-	-	-	-	-	-	3.5	48.9	1290
Menthyl acetate	-	17.4	-	-	-	-	-	-	-	-	1294
*trans*-Pinocarvyl acetate	-	-	-	-	-	0.2	-	-	-	-	1297
Carvacrol	-	-	-	-	-	-	-	-	64.5	3.5	1298
Eugenol	-	-	-	-	-	-	1.4	-	-	-	1356
Neryl acetate	-	-	0.6	-	-	2.0	-	-	-	-	1365
α-Copaene	-	-	-	-	-	-	0.4	-	-	-	1376
Geranyl acetate	-	-	0.6	-	-	3.0	-	-	-	-	1383
β-bourbonene	1.3	0.4	-	-	-	-	-	-	-	-	1384
β-Elemene	-	-	-	-	-	-	0.8	-	-	-	1391
β-Caryophyllene	0.7	0.3	0.4	-	0.4	-	0.6	2.2	2.5	3.5	1418
α- *trans*-Bergamotene	-	-	0.9	-	-	-	1.1	-	-	-	1436
α-Guaiene	-	-	-	-	-	-	1.1	-	-	-	1439
( *Z*)- β-Farnesene		0.7	-	-	-	-	-	-	-	-	1443
α-Humulene	-	-	-	-	-	-	0.5	3.4	-	0.3	1454
*trans*-β-pharnesene	-	-	-	-	43.5	-	-	-	-	-	1458
Germacrene D	0.2	0.5	-	-	0.4	-	-	-	-	0.3	1480
β-Selinene	-	-	-	-	-	-	1.0	-	-	-	1485
α-Selinene	-	-	-	-	-	-	1.7	-	-	-	1494
Bicyclogermacrene	-	1.3	-	-	5.2	-	-	-	-	-	1495
α-Zingiberene	-	-	-	-	-	-	0.6	-	-	-	1496
α-Muurolene	-	-	-	-	-	-	0.1	-	-	-	1499
*trans*-β-Guaiene	-	-	-	-	-	-	2.1	-	-	-	1500
Germacrene A	0.5	0.5	-	-	-	-	-	-	-	-	1503
β-Bisabolene	-	-	1.3	-	-	-	-	-	-	-	1509
γ-Cadinene	-	-	-	-	-	-	2.5	0.1	-	-	1513
δ-Cadinene	-	0.8	-	-	-	-	1.1	0.1	-	-	1524
*trans*-γ-Bisabolene	-	-	-	-	8.5	-	-	-	-	-	1533
*cis*-Nerolidol	-	-	-	-	-	-	0.1	-	-	-	1534
α-Cadinene	-	-	-	-	-	-	-	-	-	2.2	1538
Caryophyllene oxide	-	-	-	-	-	-	-	0.3	-	-	1581
Viridiflorol	-	0.2	-	-	-	-	-	3.0	-	-	1590
*epi*-α-Muurolol	-	-	-	-	-	-	0.4	-	-	-	1641
α-Cadinol	-	-	-	-	-	-	2.6	-	-	-	1653
Bisabolol oxide B	-	-	-	-	9.0	-	-	-	-	-	1655
Bisabolone oxide	-	-	-	-	6.0	-	-	-	-	-	1682
Chamazulene	-	-	-	-	5.6	-	-	-	-	-	1725
*cis*-Farnesol	-	-	-	-	-	-	-	-	-	-	1713
Bisabolol oxide A	-	-	-	-	8.5	-	0.2	-	-	-	1744
**Total**	**92.1**	**97.7**	**98.9**	**96.2**	**92.7**	**92.6**	**97.4**	**92.9**	**98.6**	**96.4**	

Plant abbreviations: *M.s.: Mentha spicata; M.p.: Mentha piperita; C.l.: Citrus limon; C.a.: Citrus aurantium; M.c.: Matricaria chamomilla; L.a.: Lavandula angustifolia; O.b.: Ocimum basilicum; S.o.: Salvia officinalis; O.v.: Origanum vulgare; T.v.: Thymus vulgaris*.

The results of the antibacterial activity of the essential oils are presented in Table 2 and Table 3. All the oils tested in the disc-diffusion method showed bacteriostatic activity in concentration of 1 μg/disc. The essential oils of *M*. *chamomilla *and *S*. *officinalis *exhibited the lowest antibacterial activity in the disc-diffusion method. These oils did not affect *P*. *aeruginosa *and *P*. *mirabilis*, while inhibition zones for other bacterial species were 8.0–13.0 mm and 9.0–15.0 mm, respectively. Lemon oil 9.0–19.0 mm and orange oil 8.0–19.0 mm and did not show inhibition against *P*. *aeruginosa *and *P*. *mirabilis*. The same behaviour was observed for lemon oil and orange oil. Good inhibition zones were also obtained for *M*. *spicata *and *M*. *piperita *oils, 16.0–25.0 mm and 13.0–25.0 mm, respectively. The essential oils which showed the best antibacterial activity in the disc-diffusion method were those of *T*. *vulgaris* (16.0–30.0 mm)and of *O*. *vulgare* (20.0–35.0 mm). Streptomycin at 1 μg/disc showed inhibition zones in the 0–20.0 mm range (Table 2). It can be seen that essential oils from *L*. *angustifolia, M*.* spicata*, *M*. *piperita*, *O*. *basilicum*, *O*. *vulgare *and *T*. *vulgaris *possess a higher antibacterial effect than streptomycin. Thyme and especially oregano oil showed much larger inhibition zones than other oils and streptomycin. *M*. *chamomilla *oil showed the lowest MIC (7.0–10.0 μg/mL) and MBC (8.0–15.0 μg/mL) in the microdilution method. Oils from *Citrus *species and sage oil possessed MIC at 5.0–7.5 μg/mL and MBC at 5.5–10.0 μg/mL. MIC and MBC for *L*. *angustifolia *and *O*. *basilicum *oils are very similar, 4.0–7.0 μg/mL and 4.0–9.0 μg/mL, respectively. Oils from *M*. *spicata *and *M*. *piperita *exhibited much higher antibacterial activity with the same MIC (1.0–3.0 μg/mL) and MBC (1.5–5.0 μg/mL). The essential oils from thyme and oregano inhibited all the bacteria in very small concentrations. MIC for thyme oil was 0.25–1.0 μg/mL and MBC was 0.5–1.5 μg/mL. Oregano oil possessed inhibitory effect in the range of 0.05–0.5 μg/mL, while its bactericidal effect was at 0.125–1.0 μg/mL. Streptomycin showed MIC at 1.0–3.0 μg/mL, and MBC at 1.5–5.0 μg/mL. From the obtained results it can be noticed that oils from *Citrus *species, *M*. *chamomilla*, *L*. *angustifolia*, *O*. *basilicum *and *S*. *officinalis* possessed lower antibacterial activity than streptomycin, while oils from *Mentha *species showed almost the same antibacterial potential as the antibiotic. Oregano and thyme oils showed much stronger antibacterial potential than streptomycin (Table 3).

The results of antibacterial activity of essential oil components are presented in Table 4 and Table 5. Linalyl acetate and limonene showed the lowest antibacterial activity among the components tested, with i.z. 6.0-12.0 mm; α-pinene and β-pinene possessed almost the same activity, with i.z. 8.0-16.0 mm. These three components were not effective against *P*. *aeruginosa *and *P*. *mirabilis*. Camphor inhibited bacterial growth of all bacteria and inhibition zones were 8.0-19.0 mm, linalool reacted slightly better (i.z. 8.0-20.0 mm), while 1,8-cineole showed inhibition with zones of 10.0-20.0 mm. Strong antibacterial activity was noticed for menthol (10.0-23.0 mm), thymol (18.0-30.0 mm) and especially for carvacrol (22.0-36.0 mm). Streptomycin showed activity with i.z. 0-20.0 mm, it was inactive against *L*. *monocytogenes*, *P*. *aeruginosa *and *P*. *mirabilis*. It can be seen that linalyl acetate, limonene, α-pinene and β-pinene showed lower antibacterial activity than streptomycin, while linalool, camphor and 1,8-cineole showed the same or slightly higher activity than the antibiotic. Menthol, thymol and carvacrol possessed much stronger antibacterial activity than streptomycin (Table 4). 

Linalyl acetate and limonene showed the lowest antibacterial activity also in the microdilution method, MIC at 7.0-10.0 μg/mL and MBC at 8.0-15.0 μg/mL. The monoterpenic hydrocarbons α-pinene and β-pinene also showed similar activity with MIC of 5.0-10.0 μg/mL and MBC of 5.0-13.0 μg/mL. Camphor exhibited inhibitory activity at 5.0-7.0 μg/mL and was bactericidal at 6.0-10.0 μg/mL, while linalool and 1,8-cineole showed bacteriostatic activity at 4.0-7.0 μg/mL and bactericidal at 4.0-9.0 μg/mL. Thymoland menthol showed very strong activity with MIC at 0.25-1.0 μg/mL and 0.5-3.0 μg/mL, respectively, while bactericidal effect was achieved at 0.5-1.5 μg/ml for thymol and 1.0-4.0 μg/mL for menthol. Carvacrol showed the strongest antibacterial activity with MIC at 0.02-0.5 μg/mL and MBC at 0.125-1.0 μg/mL. Only thymol, menthol and carvacrol showed higher antibacterial activity than streptomycin (MIC 1.0-3.0 μg/mL and MBC 1.5-5.0 μg/mL) (Table 5).

The essential oils investigated showed better activity against Gram-positive than Gram-negative bacteria. The antibacterial potential of oil tested in both methods can be presented as: *M*. *chamomilla *< *S*. *officinalis *< *C*. *aurantium *< *C*. *limon *< *L*. *angustifolia *< *O*. *basilicum *< *M*. *piperita *< *M*. *spicata *< *T*. *vulgaris *< *O*. *vulgare*. The essential oil of *O*. *vulgare *proved to be the most active. The antibacterial potential of essential oils’ components tested can be presented as: Linalyl acetate < limonene < β-pinene < α-pinene < camphor < linalool < 1,8-cineole < menthol < thymol < carvacrol. *Pseudomonas aeruginosa *and *Proteus mirabilis *were found to be the most resistant species; some of the essential oils and compounds were not active against them. *Micrococcus flavus* was the most sensitive bacterial species to oils and components tested.

It is obvious that hydrocarbon monoterpenes show the lowest antibacterial activity, while oxygenated compounds possess a higher potential, especially phenol-type compounds as thymol and carvacrol. It has been found [9] that oxygenated monoterpenes, exhibit strong antimicrobial activity, especially pronounced on whole cells, while hydrocarbon derivatives possess lower antimicrobial properties, as their low water solubility limits their diffusion through the medium. Hydrocarbons tend to be relatively inactive regardless of their structural type, and this inactivity is closely related to their limited hydrogen bound capacity and water solubility [10]. Ketones, aldehydes and alcohols are active, but with differing specificity and levels of activity, which is related to the present functional group, but also associated with hydrogen-bounding parameters in all cases. Previous results showed that greater antimicrobial potential could be ascribed to the oxygenated terpenes, especially phenolic compounds [11,12,13,14].

**Table 2:** Antibacterial activity of the essential oils (1.0 μg/mL) in disc-diffusion method, inhibition zones in mm.

Bacteria	*M*. *s*.	*M. p.*	*C*. *l*.	*C*. *a*.	*M*. *c.*	*L*. *a*.	*O*. *b*.	*S*. *o*.	*O*. *v*.	*T*. *v*.	Strept
***M*. *flavus***	25.0	25.0	19.0	19.0	13.0	22.0	23.0	15.0	35.0	30.0	20.0
***B*. *subtilis***	24.0	22.0	18.0	18.0	12.0	20.0	22.0	14.0	34.0	28.0	18.0
***S*. *epidermidis***	20.0	20.0	14.0	14.0	12.0	18.0	18.0	12.0	30.0	26.0	16.0
***S*. *aureus***	22.0	20.0	16.0	14.0	10.0	18.0	18.0	12.0	32.0	28.0	16.0
***S*. *enteritidis***	20.0	20.0	13.0	10.0	9.0	16.0	18.0	10.0	27.0	24.0	10.0
***S*. *typhimurium***	18.0	17.0	11.0	8.0	8.0	16.0	16.0	10.0	25.0	20.0	10.0
***E*. *coli***	16.0	16.0	12.0	9.0	9.0	14.0	14.0	10.0	26.0	22.0	12.0
***E*. *cloacae***	14.0	14.0	9.0	9.0	9.0	12.0	12.0	10.0	25.0	22.0	12.0
***P*. *mirabilis***	10.0	11.0	0	0	0	7.0	8.0	0	22.0	18.0	0
***P*. *aeruginosa***	10.0	10.0	0	0	0	6.0	8.0	0	20.0	16.0	0
***L*. *monocytogenes***	16.0	13.0	9.0	8.0	8.0	10.0	11.0	9.0	25.0	18.0	0

**Table 3:** Antibacterial activity of the essential oils (MIC and MBC - μg/mL), microdilution method.

Bacteria	*M. s.*	*M. p.*	*C.l.*	C.a.	*M.c*	*L. a.*	*O.b.*	S.o.	O.v.	T.v.	streptomycin
	MIC MBC	MIC MBC	MIC MBC	MIC MBC	MIC MBC	MIC MBC	MIC MBC	MIC MBC	MIC MBC	MIC MBC	MIC MBC
***M*. *flavus***	1.0 1.5	1.0 1.5	5.0 5.5	5.0 5.5	7.0 8.0	4.0 4.0	4.0 5.0	5.0 6.0	0.05 0.125	0.25 0.5	1.0 1.5
***B*. *subtilis***	1.5 1.5	1.5 1.5	5.0 6.0	5.0 6.0	7.0 8.0	4.0 4.5	4.0 5.0	5.56 .0	0.125 0.25	0.25 0.5	1.0 1.5
***S*. *epidermidis***	2.0 2.0	2.0 2.0	6.0 6.5	6.0 6.5	8.0 9.0	4.0 5.0	4.0 5.0	6.0 6.0	0.25 0.25	0.5 1.0	1.0 1.5
***S*. *aureus***	2.0 2.5	2.0 2.5	6.0 6.0	6.0 7.5	8.0 9.0	5.0 5.5	4.5 5.5	6.0 6.5	0.25 0.5	0.5 1.0	1.0 1.5
***S*. *enteritidis***	2.5 2.5	2.5 2.5	7.0 7.0	7.0 7.0	9.0 10.0	5.0 6.0	5.0 6.0	6.0 7.0	0.5 0.5	1.0 1.0	1.5 2.0
***S*. *typhimurium***	2.5 2.5	2.5 2.5	7.0 7.0	7.0 7.0	9.0 10.0	5.0 6.0	5.0 6.0	6.0 7.0	0.5 0.5	1.0 1.0	1.5 2.0
***E*** **. *coli***	2.5 3.0	2.5 3.0	7.5 8.0	7.5 8.0	10.0 10.0	6.0 6.0	6.0 6.0	7.0 8.0	0.5 0.5	1.0 1.5	2.0 3.0
***E*** **. * cloacae***	3.0 3.0	3.0 3.0	7.0 8.0	7.0 9.0	10.0 10.0	6.0 7.0	6.0 6.0	7.0 9.0	0.5 0.5	1.0 1.5	2.0 4.0
***P*** **. *mirabilis***	3.0 4.0	3.0 4.0	7.0 9.0	7.0 10.0	10.0 13.0	7.0 8.0	6.0 8.0	7.0 9.0	0.5 1.0	1.0 1.5	3.0 4.0
***P*** **. *aeruginosa***	3.0 5.0	3.0 5.0	7.0 10.0	7.0 10.0	10.0 15.0	7.0 9.0	6.0 9.0	7.0 10.0	0.5 1.0	1.0 1.5	3.0 5.0
***L*** **. *monocytogenes***	2.5 2.5	2.5 2.5	7.0 7.0	7.0 7.0	9.0 10.0	5.5 6.0	5.0 6.0	7.0 7.0	0.5 0.5	1.0 1.0	2.0 3.0

**Table 4:** Antibacterial activity of the components of the essential oils (1.0 μg/mL) in disc-diffusion method, inhibition zones in mm.

Bacteria	linalyl acetate	linalool	limonene	α-pinene	β-pinene	1,8-cineole	camphor	carvacrol	thymol	menthol	streptomycin
***M*. *flavus***	12.0	20.0	12.0	16.0	16.0	20.0	19.0	36.0	30.0	23.0	20.0
***B*. *subtilis***	12.0	20.0	12.0	16.0	16.0	20.0	19.0	35.0	30.0	23.0	18.0
***S*. *epidermidis***	10.0	16.0	12.0	14.0	14.0	18.0	16.0	32.0	25.0	22.0	16.0
***S*. *aureus***	10.0	16.0	10.0	14.0	14.0	18.0	18.0	32.0	22.0	20.0	16.0
***S*. *enteritidis***	8.0	16.0	9.0	12.0	10.0	16.0	14.0	29.0	22.0	18.0	10.0
***S*. *typhimurium***	8.0	14.0	8.0	10.0	8.0	16.0	13.0	27.0	22.0	18.0	10.0
***E*. *coli***	8.0	12.0	9.0	10.0	10.0	14.0	13.0	27.0	20.0	16.0	12.0
***E*. *cloacae***	8.0	12.0	9.0	9.0	9.0	14.0	12.0	27.0	20.0	14.0	12.0
***P*. *mirabilis***	0	8.0	0	0	0	8.0	8.0	24.0	18.0	10.0	0
***P*. *aeruginosa***	0	8.0	0	0	0	8.0	8.0	22.0	18.0	10.0	0
***L*. *monocytogenes***	6.0	8.0	8.0	8.0	8.0	10.0	8.0	26.0	20.0	16.0	0

**Table 5:** Antibacterial activity of the components of the essential oils (MIC and MBC - μg/mL), microdilution method.

Bacteria	linalyl acetate	linalool	limonene	α-pinene	β-pinene	1,8-cineole	camphor	carvacrol	thymol	menthol	streptomycin
***M*. *flavus***	7.0 8.0	4.0 4.0	7.0 7.0	5.0 5.0	5.0 5.5	4.0 5.0	5.0 6.0	0.02 0.05	0.25 0.5	0.5 1.0	1.0 1.5
***B*. *subtilis***	7.0 8.0	4.0 4.0	7.0 7.0	5.0 6.0	5.0 6.0	4.0 5.0	5.5 6.0	0.125 0.25	0.25 0.5	0.5 1.0	1.0 1.5
***S*. *epidermidis***	8.0 9.0	4.0 5.0	8.0 8.0	6.0 6.0	6.0 6.5	4.0 5.0	6.0 6.0	0.25 0.25	0.25 0.5	1.0 1.0	1.0 1.5
***S*. *aureus***	8.0 9.0	5.0 5.0	8.0 8.0	6.0 7.0	6.0 7.5	5.0 6.0	6.0 6.5	0.25 0.5	0.25 0.5	1.0 1.0	1.0 1.5
***S*. *enteritidis***	9.0 10.0	5.0 6.0	9.0 10.0	8.0 9.0	9.0 9.0	5.0 6.0	6.0 7.0	0.5 0.5	0.5 1.0	1.0 1.5	1.5 2.0
***S*. *typhimurium***	9.0 10.0	5.0 6.0	9.0 10.0	8.0 9.0	8.0 9.0	5.0 6.0	6.0 7.0	0.5 0.5	0.5 1.0	1.0 1.5	1.5 2.0
***E*. *coli***	10.0 12.0	6.0 7.0	10.0 12.0	8.0 10.0	8.0 10.0	6.0 8.0	7.0 8.0	0.5 0.5	1.0 1.5	1.0 2.0	2.0 3.0
***E*. *cloacae***	10.0 12.0	6.0 7.0	10.0 10.0	8.0 10.0	9.0 10.0	6.0 8.0	7.0 9.0	0.5 0.5	1.0 1.5	2.0 2.0	2.0 4.0
***P*. *mirabilis***	10.0 15.0	6.0 8.0	10.0 15.0	8.0 10.0	9.0 10.0	6.0 8.0	7.0 9.0	0.5 1.0	1.0 1.5	2.0 3.0	3.0 4.0
***P*. *aeruginosa***	10.0 15.0	7.0 9.0	10.0 15.0	10.0 12.0	10.0 13.0	7.0 9.0	7.0 10.0	0.5 1.0	1.0 1.5	3.0 4.0	3.0 5.0
***L*. *monocytogenes***	9.0 10.0	5.0 6.0	8.0 10.0	8.0 10.0	9.0 10.0	5.0 6.0	7.0 7.0	0.5 0.5	1.0 1.0	2.0 2.0	2.0 3.0

It seems evident that there is a relationship between the high activity of the *Thymus* and *Oregano *type oils and the presence of phenol components, such as thymol and carvacrol. The high antimicrobial activity of these essential oils could be explained by their high percentage of phenol components. It seems likely, that carvacrol interferes with the activity of cell wall enzymes like chitin synthase/chitinase as well as α- and β- glucanases of fungi [15,16]. Consequently, the high content of phenolic components may account for the high antifungal activity of oregano-type oils.

It can be seen that the growth of tested bacteria responded differently to the essential oils and their components, which indicates that different components may have different modes of action or that the metabolism of some bacteria is able to better overcome the effect of the oil or adapt to it. Gram negative bacteria are in general more resistant than Gram positive. Some of the oils (*Citrus *species, *M*. *chamomilla*, *S*. *officinalis*) and components (linalyl acetate, limonene, α-, β-pinene) tested in here and even more so streptomycin did not affect *P*. *aeruginosa *and *P*. *mirabilis*.

The strong antibacterial activity of some oils (*Mentha *species, *T*. *vulgaris*, *O*. *vulgare*) and their components (menthol, thymol, carvacrol) can be explained by the high percentage of these components in the oils. For the remaining oils, no significant correlation between the antibacterial activity and the percentage of the major components has been found. This suggested that the components present in the great proportions are not necessarily responsible for a great share of the total activity. The different antibacterial activity exhibited by the oils, compared with those of their major components, can be explained by either the synergistic effect of the different components in the oil and/or by the presence of other components that may be active even in small concentrations.

The MICs are generally lower for both essential oils and all the components investigated, in the disc-diffusion assays. The limitation of the oils’ activity can be explained by the low water solubility of the oil and its components which limits their diffusion through the agar medium in the disc-diffusion method. Only the more water-soluble components, such as 1,8-cineole, diffuse into the agar. The hydrocarbon components either remain on the surface of the medium or evaporate [10]. That could be the reason for the better results obtained by the microdilution method. Broth method, carried out in microtiter trays, has the advantage of lower workloads for a larger number of replicates and the use of small volumes of the test substance and growth medium. Several reports on the antimicrobial effectiveness of essential oils in food suggest that the use of oils may improve food safety [2,17,18,19]. There are also considerable changes in legislation and there are increasing consumer trends for more natural alternatives to chemical bactericides [20].

Use of essential oils is particularly advisable because herbs and spices are commonly added in food to obtain a specific taste. Of all natural antimicrobials we tested in this work the results indicate that the essential oils of *Origanum vulgare* and *Thymus vulgaris*, as well as their components, carvacrol and thymol were the most promising. Addition of various plant derived antimicrobials in combination should improve both the spectrum of activity and the level of inhibition due to synergistic effects. Thus, combination of these compounds might have even higher potential. The use of essential oils in foods as preservatives is limited, and possible reasons for this limitation may be the strong smell and taste of these substances when used at effective doses and the decrease in their effectiveness when they are added to complicated food matrices [21] compared with microbiological media. In salads and dressings, spices, which are the main source of essential oils, are part of the product formulation as flavoring agents, and thus the problem is moderated. The antimicrobial action of essential oils in model food systems or in real food is well documented in the literature [21,22,23]. Although the majority of the essential oils are classified as Generally Recognized As Safe (GRAS) [24], their use in foods as preservatives is often limited due to flavor considerations, since effective antimicrobial doses may exceed organoleptically acceptable levels. Still, there are strong consumer trends towards natural alternatives to chemical bactericides [20] and this is supported by changes in legislation. Therefore, there is an increasing demand for accurate knowledge of the minimum inhibitory (effective) concentrations (MIC) of essential oils to enable a balance between the sensory acceptability and antimicrobial efficacy [25] in the food matrix. Oregano essential oil was examined as an alternative natural additive and found to contribute to the intrinsic safety of eggplant salad, acting synergistically with low pHs and storage temperatures [21]. Additionally, concentrations of essential oil as low as 0.7% appeared to be effective and organoleptically acceptable as well. Addition of oils is therefore not problematic especially not when used in such a small amount as we define according to the MIC’s and MBC’s obtained in this paper.

## Conclusions

A large variety of commercial antibiotics and food additives are used to control infections and diseases in humans due to the consumption of spoiled food. These may cause severe hypersensitivity reactions and lead to antibiotic resistance of human pathogens. Next to the threat of drug resistance, and other infection related phenomena, there is a growing consumer demand for food that is free of chemical food additives. Further, there is increasing legislation against the use of these, especially of chemical antimicrobials. It is, therefore, necessary to develop alternative natural and safe methods for controlling bacterial and fungal infections in and through food. The goal of the present study was to develop safe, effective, and inexpensive food formulations and processes to reduce the presence of pathogens in food. The antimicrobial compounds identified in this study as the most active against major food-borne pathogens are candidates for future studies of synergism, compatibility, and activity in food or food-processing systems. They may replace conventional chemical antimicrobials. Because of their very high specific activity essential oils may be used at low and non-toxic concentrations for prevention and treatment of intestinal diseases in animals and humans caused by *E. coli*, *Salmonella*, *Listeria*, and other pathogenic bacterial species.

## Experimental

### Plant material

The aerial parts of *Matricaria chamomilla* were collected during the flowering period, May 2001, in Pančevo, Serbia. The aerial parts of *Mentha piperita* and *M. spicata* were collected in July 2001 while those of *Lavandula angustifolia*, *Ocimum basilicum *and *Thymus vulgaris* were collected in August 2001 at the experimental field of the Institute for Medicinal Plant Research “Dr Josif Pančić”, in Pančevo, Serbia. The aerial parts of *Salvia officinalis *were collected in July 2001 in Risan, Montenegro and those of *Origanum vulgare* were collected in August 2001, from the experimental field near Paraćin, Serbia. Voucher specimens for each plant were deposited in the Herbarium of the Institute of Botany and Botanical Garden, Faculty of Biology, University of Belgrade, Serbia.

### Oil isolation and analysis

Essential oils from *Lavandula angustifolia*, *Matricaria chamomilla*, *Mentha piperita*, *M. spicata*, *Ocimum basilicum* and *Thymus vulgaris* were prepared by water-distillation upon collection by the Institute of Medicinal Plant Research “Dr Josif Pančić”, Belgrade. The essential oils of *Citrus aurantium* (cat. No. 08600030) and *C. limon* (cat. No. 08600053) are commercial preparations obtained from Akras Flavours AG (Austria). All the components tested (camphor, carvacrol, 1,8-cineole, linalyl acetate, limonene, linalool, menthol, α-pinene, β-pinene, and thymol) are from the Institute of Medicinal Plant Research “Dr Josif Pančić”. Basically, dried leaves and flowering tops were ground to a powder, and 50 grams of dry material were distilled for 2 hours using a Clevenger-type apparatus. Analyses of this oil were performed by GC fitted with an FID, and GC/MS on fused silica capillary column PONA (cross-linked methyl silicone gum, 50 m × 0.2 mm, 0.5 µm film thickness). For these purposes a Hewlett-Packard, model 5890, series II gas chromatograph equipped with a split-splitless injector was used. Sample solution in ethanol (0.2%) was injected in split mode (1:100) at 250 °C. Detector temperature was 300 °C (FID), while column temperature was linearly programmed from 40°–280 °C, at a rate of 2 °C/min. In the case of GC/MS analysis, Hewlett-Packard, model 5971A MSD was used. Transfer line was kept at 280 °C. Identification of each individual compound was made by comparison of their retention times with those of pure components, matching mass spectral data with those from the Wiley library of 138,000 MS spectra. For library search PBM-based software package was used.

### Tests for antibacterial activity

The following bacterial species were used: *Bacillus subtilis *(ATCC 10707), *Enterobacter cloacae *(human isolate), *Escherichia coli* (ATCC 0157:H7), *Micrococcus flavus *(ATCC 9341),*Proteus mirabilis *(human isolate), *Pseudomonas aeruginosa* (ATCC 27853), *Salmonella enteritidis* (ATCC 13076), *S. epidermidis* (ATCC 12228) *S. typhimurium *(ATCC 13311) *Staphylococcus aureus *(ATCC 25923). The antibacterial assays were carried out by the disc-diffusion [25] and microdilution method [26,27,28] in order to determine the antibacterial activity of oils and their components against the human pathogenic bacteria. The bacterial suspensions were adjusted with sterile saline to a concentration of 1.0 × 10^5^ CFU/mL. The inocula were prepared daily and stored at +4 °C until use. Dilutions of the inocula were cultured on solid medium to verify the absence of contamination and to check the validity of the inoculum.

### Disc-diffusion test

Compounds were investigated by the disc diffusion using 4 mm filter discs. Bacteria were cultured overnight at 28 °C in LB medium and then adjusted with sterile saline to a concentration of 1.0 × 10^5^cfu/mL. The suspension was added to the top of agar (6 mL) and dissolved in Petri dishes (2 mL/agar plate) with solid peptone agar (Institute of Immunology and Virology, Torlak, Belgrade, Serbia). Filter discs with essential oils and main components (1.0 μg/mL) were placed on agar plates (1 disc per agar plate). After 24 h of incubation at 28 °C the diameter of the growth inhibition zones was measured. Streptomycin (Sigma P 7794) was used as a positive control, and 1 μL was applied to the discs from stock solution (1 mg/mL). All tests were done in duplicate; replicates were done for each oil and for each component.

### Microdilution test

The minimum inhibitory and bactericidal concentrations (MICs and MBCs) were determined using 96-well microtitre plates. The bacterial suspension was adjusted with sterile saline to a concentration of 1.0 × 10^5^cfu/mL. Compounds to be investigated were dissolved in broth LB medium (100 μL) with bacterial inoculum (1.0 × 10^4 ^cfu per well) to achieve the wanted concentrations (0.02–15.0 μg/mL). The microplates were incubated for 24 h at 28 °C. The lowest concentrations without visible growth (at the binocular microscope) were defined as concentrations that completely inhibited bacterial growth (MICs). The MBCs were determined by serial sub-cultivation of 2 μL into microtitre plates containing 100 μL of broth per well and further incubation for 72 h. The lowest concentration with no visible growth was defined as the MBC, indicating 99.5% killing of the original inoculum. The optical density of each well was measured at a wavelength of 655 nm by Microplate manager 4.0 (Bio-Rad Laboratories) and compared with a blank and the positive control. Streptomycin was used as a positive control using the same concentrations as in the disc diffusion test. Two replicates were done for each oil and each component.
